# Novel *CYP1B1* mutations in consanguineous Pakistani families with primary congenital glaucoma

**Published:** 2008-11-03

**Authors:** Sabika Firasat, S. Amer Riazuddin, Shaheen N. Khan, Sheikh Riazuddin

**Affiliations:** National Centre of Excellence in Molecular Biology, University of the Punjab, Lahore, Pakistan

## Abstract

**Purpose:**

To identify the disease-causing mutations in three consanguineous Pakistani families with multiple members affected by primary congenital glaucoma.

**Methods:**

Blood samples were collected, and DNA was extracted. Linkage analysis for reported primary congenital glaucoma loci was performed using closely spaced polymorphic microsatellite markers on genomic DNA from affected and unaffected family members. All coding exons, the exon-intron boundaries, and the 5′ untranslated region of *CYP1B1* were sequenced.

**Results:**

The alleles of chromosome 2p markers segregate with the disease phenotype in all three families with positive LOD scores. The sequencing results identified three novel mutations (L177R, L487P, and D374E) and one previously reported mutation (E229K) in *CYP1B1* that segregate with the disease phenotype in their respective families. None of these sequence variations were present in 96 ethnically matched control samples.

**Conclusions:**

These results strongly suggest that missense mutations in *CYP1B1* are most likely to be responsible for primary congenital glaucoma in these families.

## Introduction

Glaucoma is the second leading cause of visual loss and accounts for approximately 15% cases of blindness worldwide [1]. It is estimated to affect 60 million people by 2010 and predicted to rise to 80 million by 2020 [2]. Glaucoma is a group of poorly understood neurodegenerative disorders that are usually associated with elevated intraocular pressure [2]. It is clinically characterized by the degeneration of the optic nerve, loss of retinal ganglion cells, and characteristic changes in the visual field, which all lead to irreversible vision loss [3]. Although there has been much progress in finding new genes and detecting disease-related mutations, little is known about the function of the mutated gene products and the underlying pathogenic mechanisms. Further, it is estimated that all the known loci/genes of glaucoma account for a minority of all cases of glaucoma, and hence, many glaucoma genes remain to be identified.

Primary congenital glaucoma (PCG) is an inherited ocular congenital anomaly of the trabecular meshwork and anterior chamber angle [4-7]. This leads to the obstruction of aqueous outflow and increased intraocular pressure (IOP), which results in optic nerve damage leading to blindness. The disease manifests in the neonatal or early infantile period with symptoms of photophobia, epiphora, signs of enlargement of the globe, edema, opacification of the cornea, and breaks in the Descemet's membrane. The mode of inheritance is largely autosomal recessive with variable penetrance, but rare cases of pseudo dominance are also seen in families with multiple consanguinity [8-11]. Three chromosomal loci have been linked to PCG, *GLC3A* (2p21; OMIM 231300), *GLC3B* (1p36; OMIM 600975), and *GLC3C* (14q24.3) [12,13]. To date, only mutations in the human cytochrome P450 gene, *CYP1B1* (OMIM 601771), have been reported to cause PCG.

Here, we report three consanguineous Pakistani families diagnosed with early onset primary congenital glaucoma. Linkage analysis with chromosome 2p21 markers that were harboring *CYP1B1* provided positive LOD scores. Sequencing of *CYP1B1* identifies three novel mutations (L177R, L487P, and D374E) and one previously reported mutation (E229K) that segregated with the disease phenotype in their respective families. None of these sequence variations were present in 96 ethnically matched control samples.

## Methods

Thirteen consanguineous Pakistani families with PCG were recruited to participate in a study to understand the genetic aspects of glaucoma at the National Centre of Excellence in Molecular Biology (Lahore, Pakistan). This study was approved by the internal review board (IRB) of the National Centre of Excellence in Molecular Biology. The participating subjects gave informed consent, consistent with the tenets of the Declaration of Helsinki. All three families described in this study are from the Punjab province of Pakistan.

A detailed medical history was obtained by interviewing family members. All of the ophthalmological examinations including slit lamp biomicroscopy and applanation tonometry were completed at Layton Rahmatullah Benevolent Trust (LRBT) hospital (Lahore, Pakistan). Diagnosis of PCG was based on established criteria that include measurement of IOP, measurement of corneal diameters, observation of optic nerve head where possible, and symptoms of corneal edema including photophobia, buphthalmos, cloudy cornea, and excessive tearing. Patients with elevated IOP associated with other systemic or ocular abnormalities were excluded. Blood samples were collected from affected and unaffected family members. DNA was extracted by a nonorganic method described by Grimberg et al. [14]

### Genotype analysis

Genotype analysis was performed with 12 highly polymorphic fluorescent markers for *GLC3A*, *GLC3B*, and *GLC3C* loci. Briefly, each reaction was performed in 5ml reaction mixture containing 40 ng genomic DNA, 1X PCR Buffer, 1mM dNTP mix, 2.5 mM MgCl2, and 0.2 U *Taq* DNA polymerase (Ampli Taq Gold Enzyme; Applied Biosystems, Foster City, CA). Amplification was performed in a GeneAmp PCR System 9700 (Applied Biosystems). Initial denaturation was performed for 5 min at 95 °C followed by 10 cycles of 15 s at 94 °C, 15 s at 55 °C, and 30 s at 72 °C and then 20 cycles of 15 s at 89 °C, 15 s at 55 °C, and 30 s at 72 °C. The final extension was performed for 10 min at 72 °C followed by a final hold at 4 °C. Polymerase chain reaction (PCR) products from each DNA sample were pooled and mixed with loading cocktail HD-400 containing size standards (Applied Biosystems). The resulting PCR products were separated in an ABI 3100 DNA Analyzer and analyzed by using GeneMapper software packages (Applied Biosystems).

### Linkage analysis

Two-point linkage analysis was performed using the FASTLINK version of MLINK from the LINKAGE program package [15,16]. All linkage packages are provided in the public domain by the Human Genome Mapping Project Resources Centre (Cambridge, UK). Maximum LOD scores were calculated using ILINK. Autosomal recessive PCG was analyzed as a fully penetrant trait with an affected allele frequency of 0.001. The marker order and distances between the markers were obtained from the Marshfield database and the National Center for Biotechnology Information (NCBI) chromosome 2 sequence maps. Allele frequencies were estimated from 100 unrelated and unaffected individuals from the Punjab province of Pakistan.

### Mutation screening

Primer pairs for individual exons of *CYP1B1* were designed using the Primer3 program. The primers are listed in Table 1. Amplifications were performed in a 25 μl reaction containing 50 ng of genomic DNA, 2.5 μl 1X PCR buffer, 8 pmoles of each primer, 2.5 mM dNTP, 2.5 mM MgCl_2_, and 0.2 U *Taq* DNA polymerase (Applied Biosystems). Amplification was performed in a GeneAmp PCR System 9700 (Applied Biosystems). PCR amplification consisted of a denaturation step at 96 °C for 5 min, followed by 40 cycles, each at 96 °C for 45 s followed by 57 °C for 45 s and 72 °C for 1 min. PCR products were analyzed on 2% agarose gel and purified by ethanol precipitation. The PCR primers for each exon were used for bidirectional sequencing using Big Dye Terminator Ready reaction mix (Applied Biosystems) according to the manufacturer’s instructions. Sequencing products were precipitated, resuspended in 10 μl of formamide, and denatured at 95 °C for 5 min. Sequencing was performed on an ABI 3100 Automated sequencer (Applied Biosystems). Sequencing results were assembled using the ABI PRISM sequencing analysis software version 3.7 and analyzed using Chromas software version 1.45.

**Table 1:** The primer sequences and annealing temperatures for *CYP1B1*.

**Exon**	**Forward Primer**	**Reverse Primer**	**Annealing Temp (°C)**
1	GCTCCCATGAAAGCCTGCTG	ACGCCACCCGCTACCTGTAA	63
2a	GGCCATTTCTCCAGAGAGTC	GAACTCTTCGTTGTGGCTGA	57
2b	ATGATGCGCAACTTCTTCAC	CACTGTGAGTCCCTTTACCG	57
3a	AGCCTATTTAAGAAAAAGTGGAA	AATTGAGAAGCAGCACAAAA	54
3b	ATAAGAAGCAAGAGGCAAGC	AGGTACAACATCACCTTGGAG	55
3c	CAGTTGCTCAAAAAGAAATCA	AAAGAACATCCAGGTAATTCA	55
3d	TGAACATTCTCCTGTGGAAG	ATTCCAAACCACAAAACAGA	54
3e	TTTGGAGCACAAAATTCAAA	AGCTTTGACATACAAATGAAGC	55
3f	TGCTGACAACCATTAAAGTCA	AAATGTAACCTCCGTGTTGG	55

## Results

All three families reported here, PKGL021, PKGL022, and PKGL026, are from the Punjab province of Pakistan. A detailed medical history was obtained by interviewing family members. Ophthalmological examinations were completed at the LRBT hospital in Lahore, Pakistan. The symptoms of glaucoma in affected individuals in family PKGL021 were present at birth. Visual acuity is confined to hand motion or light perception. The IOP is controlled with antiglaucoma treatments. Similarly, glaucoma in affected individuals of family PKGL022 was diagnosed within the first month after birth. Clinical features include bilateral buphthalmos eyes, corneal opacity, and central corneal haze. Visual acuity was reduced to counting fingers. The IOP of both affected individuals of PKGL022 was considerably higher than the affected individuals of PKGL021 and PKGL026. The clinical records indicate that individual 11 and 12 have 43/50 (OD/OS), and 23/37 (OD/OS) mm Hg of intraocular pressure, respectively. Lastly, ophthalmic examinations of affected individuals of PKGL026 show typical features of glaucoma with elevated IOP and visual acuity reduced to counting fingers or light perception. The symptoms of glaucoma were either present at birth or appeared in the first six weeks after birth.

**Figure 1:**
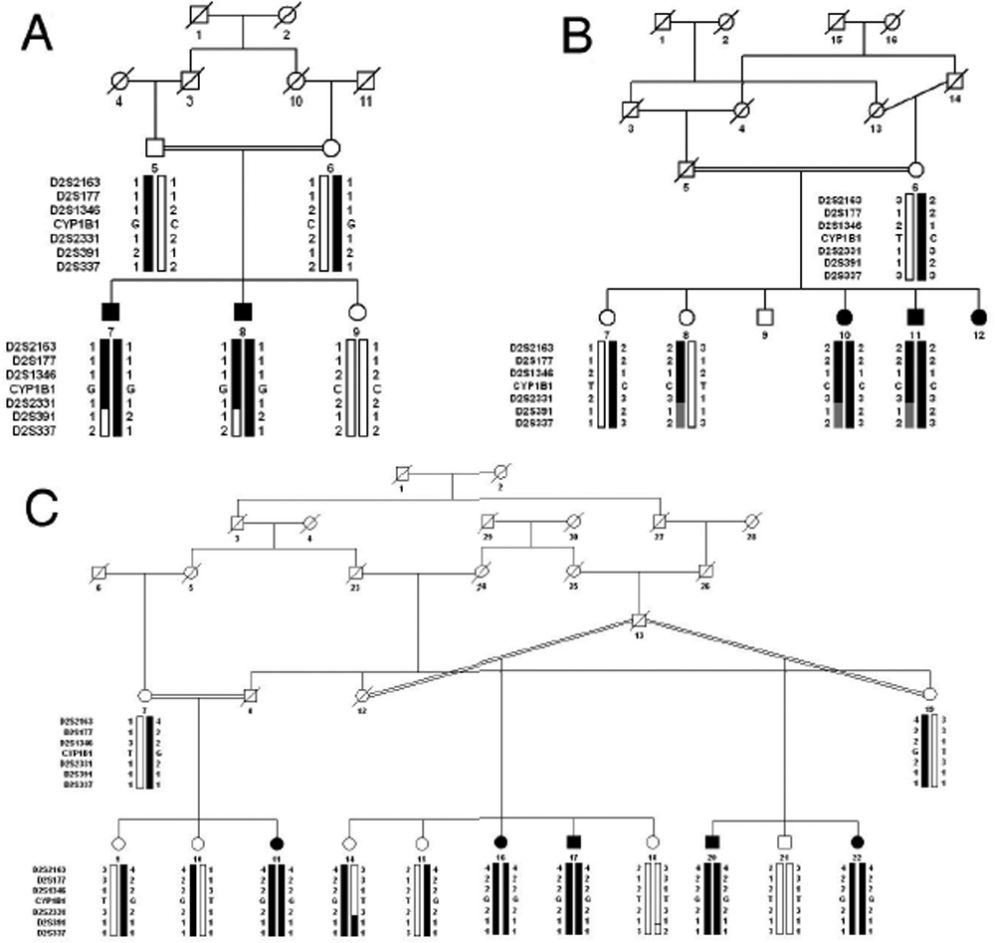
Family pedigrees. Pedigrees of **A**) PKGL021, **B**) PKGL022 and **C**) PKGL026. Squares denote males while circles denote females. Filled symbols indicate affected individuals. A double line between individuals signifies consanguinity, and a diagonal line through a symbol indicates a deceased family member. The haplotypes of six adjacent chromosome 2p21 microsatellite markers are shown with alleles forming the risk haplotype shaded black, alleles cosegregating with PCG but not showing homozygosity shaded gray, and alleles not cosegregating with PCG shown in white.

Two-point linkage analysis with chromosome 2p21 markers provided positive LOD scores. For PKGL021, the maximum LOD scores of 1.43, 1.47, and 0.91 were obtained with markers D2S352, D2S1346, and D2S2331 at θ=0, respectively (Table 2). Similarly, for PKGL022, maximum LOD scores of 1.65, 1.35, 1.48, and 1.60 were obtained with markers D2S2163, D2S177, D2S1346, and D2S2331 at θ=0, respectively (Table 2). Lastly, for PKGL026, maximum LOD scores of 5.16, 4.86, 3.71, and 2.96 were obtained with markers D2S2163, D2S177, D2S1346, and D2S2331 at θ=0, respectively (Table 2). Haplotype analysis show that all the affected individuals of PKGL021, PKGL022, and PKGL026 have homozygous alleles for D2S2163, D2S177, D2S1346, and D2S2331 short tandem repeat (**STR**) markers whereas the normal individuals are either heterozygous carriers of the disease allele or are homozygous for the normal allele (Figure 1).

**Table 2:** Two-point parametric LOD scores of PKGL021, PKGL022, and PKGL026 with chromosome 2p markers.

**Marker**	**cM**	**Mb**	**0.00**	**0.01**	**0.03**	**0.05**	**0.07**	**0.09**	**0.10**	**0.20**	**0.30**	**Z_max_**	**θ_max_**
**PKGL021**													
D2S149	34.0	14.31	−4.34	−1.19	−0.74	−0.54	−0.42	−0.33	−0.30	−0.12	−0.06	−0.06	0.30
D2S2150	40.5	20.39	−2.59	−1.27	−0.83	−0.64	−0.52	−0.44	−0.41	−0.23	−0.17	−0.17	0.30
D2S352	50.7	31.34	1.43	1.39	1.33	1.26	1.19	1.12	1.09	0.73	0.39	1.43	0.00
D2S2163	59.4	37.78	0.38	0.37	0.34	0.32	0.29	0.27	0.25	0.14	0.06	0.38	0.00
D2S177	59.4	37.87	0.43	0.42	0.39	0.36	0.33	0.31	0.29	0.17	0.07	0.43	0.00
D2S1346	59.4	38.11	1.47	1.44	1.37	1.3	1.24	1.17	1.13	0.79	0.46	1.47	0.00
D2S2331	59.9	38.79	0.91	0.89	0.82	0.77	0.71	0.65	0.62	0.34	0.09	0.91	0.00
D2S391	70.3	46.25	−2.89	−1.27	−0.82	−0.61	−0.48	−0.39	−0.35	−0.13	−0.05	−0.05	0.30
D2S337	80.7	61.51	−2.89	−1.27	−0.82	−0.61	−0.48	−0.39	−0.35	−0.13	−0.05	−0.05	0.30
**PKGL022**													
D2S149	34.0	14.31	0.16	0.14	0.13	0.12	0.12	0.11	0.11	0.09	0.04	0.16	0.00
D2S2150	40.5	20.39	0.48	0.46	0.43	0.40	0.37	0.34	0.32	0.18	0.08	0.48	0.00
D2S352	50.7	31.34	0.16	0.14	0.13	0.12	0.12	0.11	0.11	0.09	0.04	0.16	0.00
D2S2163	59.4	37.78	1.65	1.61	1.52	1.43	1.35	1.26	1.22	0.79	0.40	1.65	0.00
D2S177	59.4	37.87	1.35	1.31	1.23	1.16	1.08	1.01	0.97	0.61	0.30	1.35	0.00
D2S1346	59.4	38.11	1.48	1.44	1.36	1.28	1.20	1.12	1.08	0.69	0.34	1.48	0.00
D2S2331	59.9	38.79	1.60	1.56	1.48	1.39	1.31	1.22	1.18	0.76	0.38	1.60	0.00
D2S391	70.3	46.25	−3.92	−1.49	−0.98	−0.73	−0.57	−0.45	−0.40	−0.13	−0.04	−0.04	0.30
D2S337	80.7	61.51	−3.08	−1.03	−0.59	−0.39	−0.28	−0.20	−0.17	−0.02	0.01	0.01	0.30
**PKGL026**													
D2S149	34.0	14.31	-∞	−2.82	−1.08	−0.36	0.05	0.32	0.41	0.76	0.62	0.76	0.20
D2S2150	40.5	20.39	-∞	−4.62	−2.80	−2.00	−1.50	−1.14	−1.00	−0.22	0.05	0.05	0.30
D2S352	50.7	31.34	-∞	−1.55	−0.68	−0.31	−0.10	0.04	0.10	0.30	0.25	0.30	0.20
D2S2163	59.4	37.78	5.16	5.06	4.86	4.66	4.45	4.24	4.14	3.06	1.97	5.16	0.00
D2S177	59.4	37.87	4.86	4.76	4.56	4.36	4.16	3.96	3.86	2.83	1.79	4.86	0.00
D2S1346	59.4	38.11	3.71	3.71	3.65	3.56	3.44	3.3	3.23	2.42	1.56	3.71	0.00
D2S2331	59.9	38.79	2.96	2.92	2.82	2.71	2.59	2.47	2.41	1.77	1.15	2.96	0.00
D2S391	70.3	46.25	1.60	1.57	1.49	1.41	1.33	1.25	1.21	0.81	0.45	1.60	0.00
D2S337	80.7	61.51	-∞	1.52	1.85	1.92	1.92	1.87	1.84	1.35	0.73	1.92	0.05

LOD scores were calculated at different θ values for each marker with the FASTLINK version of MLINK from the LINKAGE program package. Maximum LOD scores for each marker were calculated using ILINK.

**Figure 2:**
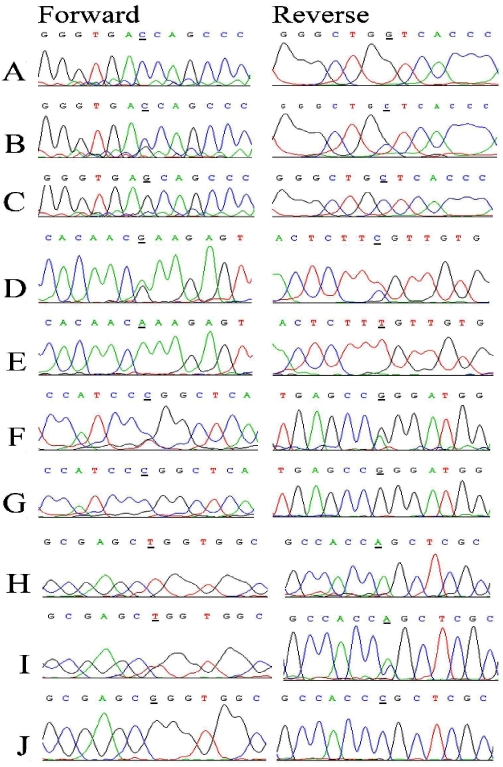
Sequence chromatograms. The forward and reverse sequence chromatograms of (**A**) unaffected individual 9 of PKGL021, (**B**) individual 5 of PKGL021, heterozygous and (**C**) individual 7 of PKGL021, homozygous for a C→G transversion in exon 3, c.1122C>G, resulting in a; p.D374E, (**D**) individual 8 of PKGL022, heterozygous and (**E**) individual 10 of PKGL022, homozygous for G→A transition in exon2, c.685G>A, resulting in mutation p.E229K, (**F**) individual 8 of PKGL022, heterozygous and (**G**) individual 10 of PKGL022, homozygous for a T→C transition in exon 3: c.1460T>C resulting in a; p.L487P (**H**) unaffected individual 21 of PKGL026, (**I**) individual 7 of PKGL026, heterozygous, and (**J**) individual 16 of PKGL026, homozygous for a T→G transversion in exon 2: c.530T>G: resulting in p. L177R.

All coding exons, exon-intron boundaries, and the 5′ untranslated region of *CYP1B1* were sequenced in all three families. In PKGL021, a C→G transversion in exon 3, c.1122C>G, was identified resulting in an aspartic acid to glutamic acid change, D374E (Figure 2). In PKGL022, a G→A transition in exon 2, c.685G>A, was identified resulting in a glutamic acid to lysine change, E229K (Figure 2). Additionally, in PKGL022, a T→C transition in exon 3, c.1460T>G, was identified leading to a leucine to proline change, p. L487P (Figure 2). Both these mutations segregated with the disease phenotype in PKGL022. Finally, in PKGL026, a T→G transversion in exon 2, c.530T>G, was identified leading to a leucine to arginine change, L177R (Figure 2). All the above mentioned variations were homozygous and segregated with the disease phenotype in their respective families. None of these sequence variations were present in 96 ethnically matched control samples. Six single nucleotide polymorphisms (SNPs) including rs2617266, a SNP residing 12 bp upstream of the first coding exon of *CYP1B1*, rs10012, rs1056827, rs1056836, rs1056837, and rs1800440 were examined to construct the disease haplotype. As shown in Table 3, the affected individuals of PKGL021 and PKGL022 harbor T-G-T-C-C-A haplotype whereas PKGL026 has a C-C-G-G-T-A haplotype.

**Table 3:** Single nucleotide polymorphism (SNP) profile of PCG patients from all three families linked to *CYP1B1*.

**Family**	rs2617266	rs10012	rs1056827	rs1056836	rs1056837	rs1800440
PKGL021	T	G	T	C	C	A
PKGL022	T	G	T	C	C	A
PKGL026	C	C	G	G	T	A

To trace *CYP1B1* mutations identified in this study haplotypes for six intragenic SNPs including rs2617266, which is 12 bp upstream of the first coding exon of *CYP1B1*, rs10012, rs1056827, rs1056836, rs1056837, and rs1800440 were constructed.

## Discussion

**Figure 3:**
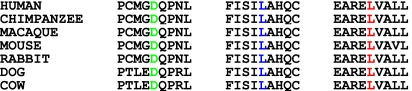
Sequence alignment of amino acids of CYP1B1 among higher primate species. The alignment of CYP1B1 among higher primate species shows the conservation of Asp374 (green), Leu487 (blue), and Leu177 (red).

Here, we report three consanguineous Pakistani families diagnosed with primary congenital glaucoma. Linkage analysis with chromosome 2p21 markers, which harbor *CYP1B1*, provided positive LOD scores. Sequencing of *CYP1B1* identified three novel mutations (L177R, L487P, and D374E) and a previously reported mutation (E229K) in these families. All variations segregated with the disease phenotype in the respective families, and none of them were present in 96 control samples of similar ethnic population. Amino acids, Leu177, Asp374, and Leu487, are highly conserved among higher primate species (Figure 3). Linkage to chromosome 2p21 harboring *CYP1B1,* segregation of these variations with the disease phenotype, and the absence of these variations in 96 control samples of similar ethnic population strongly suggest that these variations are most likely to be responsible for primary congenital glaucoma in these families.

*CYP1B1* is a member of the CYP450 super family, which contains 58 functional genes in the human genome [2]. The gene product is a 543 amino acid protein that contains the NH_2_-terminal membranous region; a 10 residue long, proline-rich region; and a cytosolic globular domain [2]. Mutations in *CYP1B1* are the predominant cause of PCG in patients from various ethnic backgrounds. Previous studies have shown that missense mutations in *CYP1B1* affect highly conserved and functionally important regions of CYP1B1, resulting in significant structural changes and reduced CYP1B1 activity [11,17-21].

In PKGL021, we identified a homozygous missense change that substitutes aspartic acid at position 374 with glutamic acid in affected individuals. Previously, aspartic acid to asparagine substitution at the same position has been reported to lead to PCG phenotype in Saudi Arabian patients [22]. Amino acid 374 maps to helix K, one of the highly conserved core structures thought to be involved in correct folding and heme binding of the cytochrome P450 molecule [23]. Further mutations that map to helix K are reported to be responsible for the severe PCG phenotype in Indian patients [21].

In PKGL022, a leucine to proline substitution at residue 487 and a previously reported mutation, E229K, segregated in an autosomal recessive pattern with the disease phenotype. L487P affects the highly conserved position marking the end of helix L, and the proline substitution at this position could potentially disrupt the helical structure and possibly affect the native three-dimensional structures. Conversely, glutamic acid 229 resides in the vicinity of the substrate-binding region (SBR) and is reported to cause conformational changes in CYP1B1 [24]. Heterozygous carriers of this mutation develop PCG and primary open-angle glaucoma phenotype [25-32]. Interestingly, compound heterozygous carriers of E229K and L487P in PKGL022 show no symptoms of PCG. Noteworthy, the affected individuals of PKGL022 have higher IOP compared with affected individuals examined during this study. Mutation L177R was detected in PKGL026. Amino acid 177 in CYP1B1 resides in a highly conserved NH_2_-capping region [21]. The non-conservative replacement of a non-polar amino acid, leucine, with positively charged arginine is most likely to impair the native protein structure and consequently affect the protein function.

SNPs, especially rs2617266, rs10012, rs1056827, rs1056836, rs1056837, and rs1800440, have been used to construct haplotypes of *CYP1B1* mutations [18,33,34]. According to the proposed hypothesis of evolution, the T-G-T-C-C-A and C-C-G-G-T-A haplotypes are ancestral human haplotypes whereas the T-G-T-C-C-A haplotype is comparatively recent [35,36]. To date, approximately 50% of known *CYP1B1* mutations are associated with the C-C-G-G-T-A haplotype while the T-G-T-C-C-A and C-C-G-C-C-G haplotypes are less frequent and are associated with 9.7% and 7% of *CYP1B1* mutations, respectively [31,33,37]. Affected individuals of PKGL021 and PKGL022 harbor a T-G-T-C-C-A haplotype, which is indicative of their common ancestry. Noteworthy, the previously reported mutation, E229K, which segregates in PKGL022, has been associated with the T-G-T-C-C-A haplotype in French, German, Indian, and Iranian patients [35,36]. The D374N mutation found in Saudi Arabian patients is associated with the C-C-G-G-T-A haplotype, which is in contrast to the T-G-T-C-C-A haplotype coupled with D374E mutation in PKGL021 [38]. Finally, the L177R mutation detected in PKGL026 is present in the C-C-G-G-T-A haplotype, which is frequently associated with *CYP1B1* mutations.

Studies of pathogenic sequence variants in *CYP1B1* will contribute to better understanding of primary congenital glaucoma. Identification of these mutations reaffirms the diverse allelic heterogeneity of *CYP1B1* in the pathogenesis of PCG. This will further help in the elucidation of the structure-function relationship of CYP1B1 and hence, lead to the development of novel therapeutic approaches. Consequently, the association of specific haplotypes with pathogenic mutations will contribute to our knowledge of haplotype clustering of PCG associated with *CYP1B1* mutations. This will overall enhance our understanding of primary congenital glaucoma at the molecular level.
